# Editorial: Detection and drug treatment of emerging viral diseases

**DOI:** 10.3389/fcimb.2025.1606637

**Published:** 2025-04-16

**Authors:** Wenqiang Wei, Yuhao Li, Fusheng Si

**Affiliations:** ^1^ School of Basic Medical Sciences, Henan University, Kaifeng, Henan, China; ^2^ Department of Molecular Microbiology, Washington University School of Medicine, St. Louis, MO, United States; ^3^ Institute of Animal Science and Veterinary Medicine, Shanghai Academy of Agricultural Sciences, Shanghai, China

**Keywords:** infectious diseases, pathogen detection, vaccine development, machine learning, molecular epidemiology

## Introduction

Emerging and re-emerging pathogenic infections continue to pose a significant threat to global public health, agriculture, and economic stability. These diseases, responsible for millions of deaths annually, urgently require advancements in rapid detection methods and effective therapeutic strategies (Gharbi et al., 2025). The ongoing COVID-19 pandemic has underscored the critical importance of robust diagnostic tools and potent antiviral treatments for the efficient management and control of novel pathogens (Kevadiya et al., 2021). Typically, the rapid spread of infectious agents is driven by inadequate population immunity and the absence of effective therapeutic interventions (Baker et al., 2022). Successfully addressing these challenges requires an integrated, multidisciplinary approach that encompasses virology, immunology, epidemiology, and computational biology (Al Meslamani et al., 2024).

This Research Topic, “Detection and Drug Treatment of Emerging Viral Diseases,” brings together four pivotal studies that significantly advance our understanding of emerging viral and bacterial diseases. These studies focus on critical areas, including pathogen detection, epidemiological surveillance, vaccine development, and novel therapeutic strategies. They encompass viral and bacterial pathogens that affect both humans and animals, emphasizing the interconnectedness of health within the One Health framework. By integrating cutting-edge technologies, from multiplex diagnostics to machine learning-driven vaccine design, these studies collectively address critical gaps in pandemic preparedness and disease management, providing essential insights to improve global health outcomes.

## Advancing detection methods for bovine diarrhea viruses

The concept of “One Health” highlights the interconnectedness of human and animal health and the importance of controlling zoonotic and animal diseases to safeguard public health (Si et al., 2024; Tian et al., 2025). Rapid and accurate pathogen identification is crucial for containing infectious disease outbreaks and mitigating economic losses. A study by Yang et al. exemplifies this principle through their development of a one-step multiplex reverse-transcription quantitative real-time PCR (mRT-qPCR) assay designed to simultaneously detect three key enteric viral pathogens in calves: bovine kobuvirus (BKoV), bovine astrovirus (BoAstV), and bovine torovirus (BToV). Calf diarrhea, a major economic burden in the cattle industry, frequently involves complex co-infections that complicate diagnosis and treatment. Traditional single-pathogen assays are labor-intensive and insufficient for comprehensive surveillance. The mRT-qPCR method developed by Yang et al. demonstrates remarkable sensitivity (detection limit: 24 copies/mL) and specificity, with coefficients of variation below 1.5% and strong linear correlations (R² > 0.996), ensuring reliability and reproducibility in both clinical and research contexts. Validation using 80 clinical samples from dairy farms in Shanghai revealed specific regional prevalence patterns, with BKoV identified as the predominant pathogen (28.75%), followed by BoAstV (8.75%) and BToV (3.75%). This study not only provides the first epidemiological data on these viruses in Shanghai but also establishes a scalable model for multiplex diagnostics in resource-limited settings. Such innovations are critical for early outbreak detection and containment, aligning with global efforts to enhance agricultural resilience and food security.

## Epidemiological insights into herpesvirus infections in children

Effective surveillance and early intervention are critical to controlling herpesvirus infections in pediatric populations. Wei et al. provide valuable epidemiological data on three herpesviruses—Herpes simplex virus type 2 (HSV-2), Epstein-Barr virus (EBV), and Cytomegalovirus (CMV)—among children in Nanjing, China, spanning from 2018 to 2023. By analyzing 21,210, 49,494, and 32,457 outpatient and inpatient samples, respectively, the authors identified significant trends in herpesvirus prevalence. Overall detection rates were found to be 0.32% for HSV-2, 14.99% for EBV, and 8.88% for CMV, accompanied by a decline in incidence over the study period. Of note, the study revealed age-specific prevalence patterns: HSV-2 predominated in children aged 1-3 years, EBV was most prevalent in children aged 3-7 years, and CMV primarily affected infants aged between 28 days and 1 year. These findings underscore the importance of age-specific surveillance strategies and targeted interventions to mitigate the impact of herpesvirus infections among children.

## B-cell epitope mapping on FAdV-4 fiber-1: a leap toward subunit vaccines

Identifying antigenic epitopes is essential for the development of effective subunit vaccines and targeted therapies (Li et al., 2025). The study by Chai et al. focuses on Fowl Adenovirus Serotype 4 (FAdV-4), a major pathogen causing hepatitis-hydropericardium syndrome (HHS), which results in substantial economic losses in the poultry industry. Using a prokaryotic expression system, Chai et al. successfully expressed and purified the fiber-1 knob (F1K) protein and generated monoclonal antibodies (mAbs) by immunization of BALB/c mice. Through comprehensive immunoassays, the authors identified three novel linear B-cell epitopes—^319^SDVGYLGLPPH^329^, ^328^PHTRDNWYV^336^, and ^407^VTTGPIPFSYQ^417^—within the knob domain. Structural analysis using PyMOL revealed that two of these epitopes were surface-exposed on the knob trimer, while the third was internally positioned. This study not only pioneers epitope mapping on FAdV-4 fiber-1 but also lays the groundwork for subunit vaccines and diagnostics. Future applications include the development of multi-epitope vaccines or monoclonal antibody therapies as alternatives to traditional inactivated vaccines, thereby reducing the economic impact on the poultry industry.

## Machine learning approaches for salmonella vaccine development

Despite significant advancements in antiviral drug discovery, vaccination remains the gold standard for infectious disease prevention. The emergence of SARS-CoV-2 and the subsequent global COVID-19 vaccine development have underscored the importance of innovative vaccine design strategies (Chavda and Apostolopoulos, 2022). In this context, the study by Spiga et al. explores the application of machine learning in predicting immunogenic proteins for Salmonella vaccine development. The authors developed SHASI-ML, a computational framework that uses the Extreme Gradient Boosting (XGBoost) algorithm to predict immunogenic proteins in Salmonella species. Trained on a curated dataset of experimentally validated immunogenic and non-immunogenic proteins, the model achieved 89.3% precision and 91.2% specificity. Applying SHASI-ML to the Salmonella enterica serovar Typhimurium proteome, the researchers identified 292 novel immunogenic protein candidates. This study illustrates the potential of machine learning to accelerate vaccine development by prioritizing promising candidates early in the research process, thereby reducing experimental costs and time constraints. Future adaptations of this approach could extend to viral pathogens, such as influenza or coronaviruses, where rapid antigenic drift necessitates agile vaccine updates.

## Broader implications and future directions

The studies in this Research Topic collectively highlight the importance of integrating technological advancements with epidemiological and immunological research to combat emerging infectious diseases. The epidemiological insights from Wei et al. emphasize the importance of long-term surveillance and age-specific interventions in the management of pediatric herpesvirus infections. The multiplex detection platform by Yang et al. highlights technological advancements that enable rapid and cost-effective diagnosis of multiple pathogens simultaneously, which is crucial for effective disease control in agricultural contexts. The machine learning approach by Spiga et al. represents the growing role of computational biology in accelerating vaccine development against bacterial pathogens with significant public health impact.

Future research should continue to integrate epidemiological insights, advanced detection technologies, and computational modeling to develop comprehensive strategies for emerging disease management. The extension of machine learning approaches to viral pathogens holds great promise for transforming vaccine development. Additionally, the standardization of multiplex detection methods for human pathogens would enhance clinical diagnostics and response capabilities during outbreaks. As emerging viral diseases continue to threaten global health security, these studies provide an important foundation for more effective detection, surveillance, and intervention strategies.

## Conclusion

This Research Topic showcases innovative approaches across multiple domains of microbial disease research—epidemiology, diagnostic development, and computational vaccine design. Enhancing capabilities in these critical areas brings us closer to achieving effective detection, prevention, and treatment of emerging diseases. By integrating traditional research methodologies with state-of-the-art technologies, these studies contribute to the broader goal of improving global health outcomes and pandemic preparedness. Moving forward, a multidisciplinary approach, uniting virology, immunology, bioinformatics, and epidemiology, will be essential to address future infectious disease challenges.
